# An overview of invasive micropapillary carcinoma of the breast: past, present, and future

**DOI:** 10.3389/fonc.2024.1435421

**Published:** 2024-11-15

**Authors:** Pu Qiu, Qiuxia Cui, Shengchao Huang, Yuanqi Zhang, Haitao Zhang, Hui Luo

**Affiliations:** ^1^ Marine Biomedical Research Institute, Guangdong Medical University, Zhanjiang, China; ^2^ Department of Breast Surgery, Affiliated Hospital of Guangdong Medical University, Zhanjiang, China; ^3^ Department of Breast Surgical Oncology, National Cancer Center/National Clinical Research Center for Cancer, Chinese Academy of Medical Sciences and Peking Union Medical College, Shenzhen, China

**Keywords:** breast, invasive micropapillary carcinoma, poor prognosis, opportunities, challenges

## Abstract

Invasive micropapillary carcinoma of the breast (IMPC) exhibits a unique micropapillary structure and “inside-out” growth pattern. Despite its extremely low incidence, IMPC has attracted considerable attention owing to its poor prognosis. Since Siriaunkgul and Tavassoli first proposed the term IMPC in 1993 to describe its morphological characteristics, with tumor cell clusters arranged in a pseudopapillary structure within the glandular cavity, its diagnostic rate has substantially increased. Based on the in-depth study of IMPC, a more comprehensive understanding of its epidemiology, clinicopathological features, and diagnostic criteria has been achieved in recent years. The pathogenesis and specific therapeutic targets of IMPC remain unclear. However, numerous studies have delved into its high-risk biological behavior. This review discusses the opportunities and challenges associated with IMPC.

## Background

Invasive micropapillary carcinoma (IMPC) is a rare type of breast cancer with distinct histological and biological features. Morphologically, IMPC is composed of nested, morula-like, or pseudopapillary structures of neoplastic clusters devoid of fibrovascular cores surrounded by clear stromal spaces (1). In addition, micropapillary pattern (MP), associated with aggressive biological behavior and poor prognosis (2–4), has been reported in several organs, such as the lung (5), bladder (6), pancreas (7), alimentary tract (8, 9), salivary gland (10), thyroid (11), ovary (12), cervix (13), and kidney (14).

**Figure 1:**
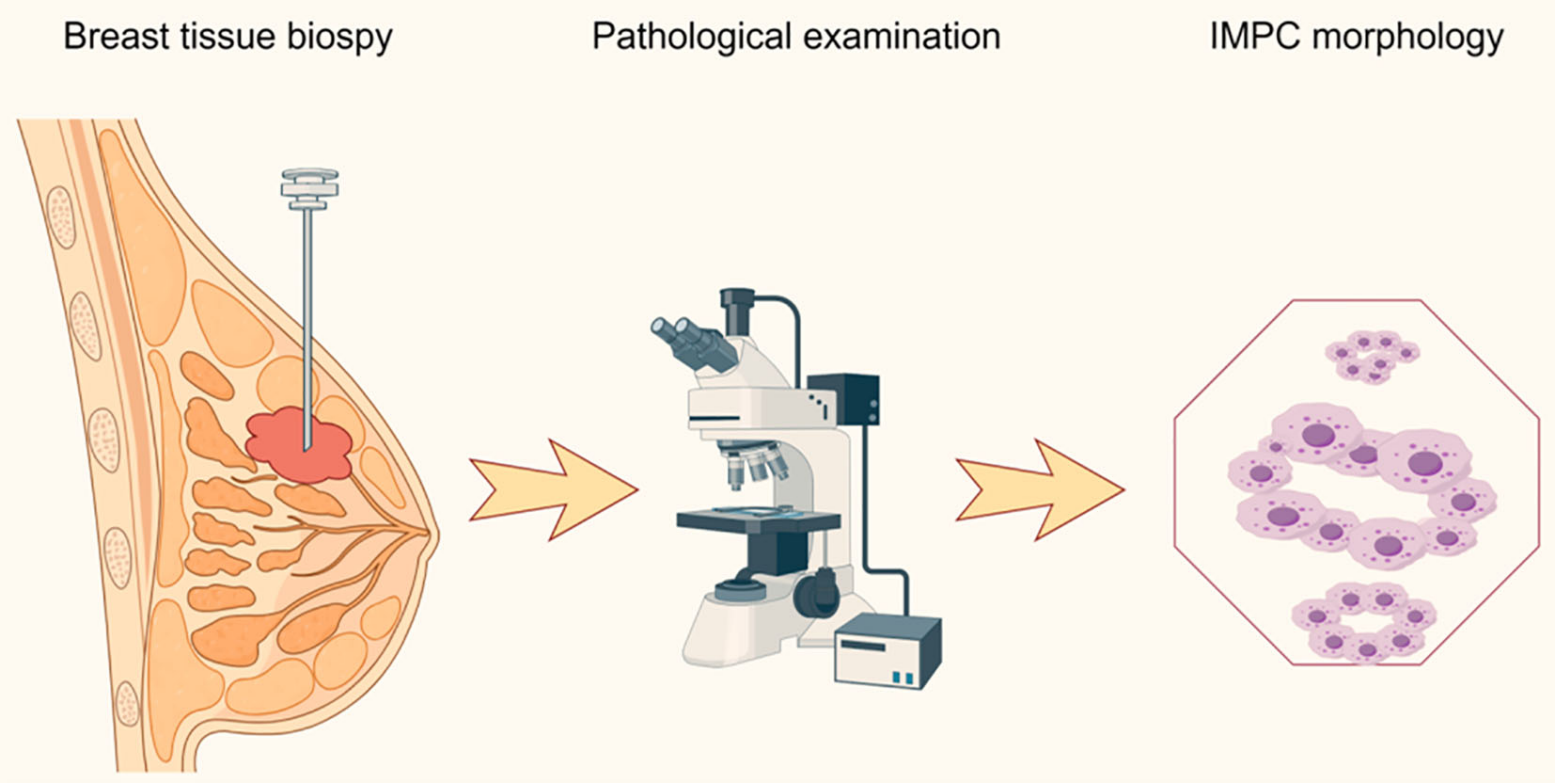
Pathological diagnostic procedure of invasive micropapillary carcinoma. IMPC, invasive micropapillary carcinoma of the breast.

In 1980, Fisher et al. first described “the exfoliative appearance structure” in breast tissue (15). Later, in 1993, Siriaunkgul and Tavassoli first proposed the term “invasive micropapillary carcinoma of the breast” and provided a detailed description of its histological features (16). Until 2003, the World Health Organization (WHO) classified IMPC as a special histological subtype of breast cancer, a classification still in use today (17–19). IMPC accounts for 2%–8% of all breast cancers (4, 19–21). However, its occurrence is usually associated with lymph node metastasis (LNM), lymphovascular invasion (LVI), and locoregional recurrence (LRR) (22, 23). Through the in-depth study of IMPC, a more comprehensive understanding of its epidemiology, clinicopathological features, and diagnostic criteria has been achieved in recent years (**Figure 1**).

Nevertheless, the high-risk biological behavior of IMPC, including its biological hallmarks, pathogenesis, and specific therapeutic targets, remains unclear. Therefore, individualized treatment for this subtype of breast cancer has not been improved. This review focuses on the research progress of IMPC and discusses its opportunities and challenges.

## Pathological features of IMPC

### Histological features

Cancerous cells typically have serrated edges and granular or eosinophilic cytoplasm when viewed under an optical microscope. Within nests, these cells form mulberry-like clusters and pseudopapillary structures, which are notable for the absence of endothelial cells and fibrovascular cores. Cancerous cells are typically enveloped by transparent fibrocollagen stromal space, resembling a swimming ring floating in the sea (17). Under an electron microscope, microvilli can be observed on the outer surface of cancerous cells, exhibiting secretory activity within the surrounding stroma (24). Furthermore, under a multiphoton microscope, six different morphologies of IMPC clusters can be detected: mulberry-like, chrysanthemum-like, tubular glandular, circular cluster, elliptical cluster, and irregular masses (25).

Invasive mucinous carcinoma or neuroendocrine carcinoma may also be accompanied by micropapillary (MP) features that are difficult to distinguish from IMPC. However, compared with pure invasive mucinous carcinoma or neuroendocrine carcinoma, the presence of MP features, such as LNM, LVI, and deteriorated differentiation, increases the probability of poor prognosis (22, 26–29). Pareja et al. reported that there was no significant genomic difference between IMPC and mucinous carcinoma with MP features. Both carcinomas exhibited recurrent gains in 1q, 6p, 8q, and 10q, along with recurrent losses in 16q, 11q, and 13q. Additionally, recurrent 8p12–8p11.2 amplification was observed in *FGFR1* (30).

Previously, the diagnostic criteria for the proportion of IMPC have not been clearly defined because of its rarity as a pure form, often accompanied by invasive ductal carcinoma (IDC) component (31). Thus, three different classification methods of pure IMPC have been proposed: MP proportion > 50%, MP proportion > 75%, and MP diameter > 5 mm. Subsequently, studies have shown that the poor prognosis of IMPC is only related to the presence of MP features rather than their proportion (32–37). Based on these findings, Fu et al. proposed that IMPC can be diagnosed as long as MP features are present in IDC and their proportion should be noted in the pathological report (38). However, pure IMPC is a rare phenomenon, found in approximately 1%–2% of breast carcinomas. The authors suggest a cutoff percentile value of 75% IMPC component to histologically identify a carcinoma as pure IMPC (39).

When distributed focally, IMPC mostly exhibits the “inside-out” growth pattern and reversal of neoplastic cell polarity, distinguishing it from other breast cancer subtypes (40). Cytologically, the inside-out growth pattern induces a reversal of cell polarity, causing the apical surface of cells to face the stroma instead of the lumen. This alteration confers highly invasive and metastatic potential (41). Furthermore, the “inside-out” growth pattern can be confirmed through staining for MUC1/epithelial membrane antigen (EMA), sialyl Lewis X, p120 catenin, or Annexin A2 on the cell membrane (42–44). Nonetheless, their staining emerges almost exclusively on the peripheral membrane, with no apical staining, also known as “goblet staining” (45).

### Immunohistochemical features

Among the molecular subtypes of IMPC, luminal subtypes account for about 70%−80%, while *HER2*-overexpressing and triple-negative subtypes account for only 10%−20% (46, 47). However, the criteria for evaluating the *HER2* status of IMPC are controversial. According to the 2018 American Society of Clinical Oncology and College of American Pathology (ASCO/CAP) *HER2* testing recommendations, moderate to intense incomplete membrane staining of *HER2* can be scored as 2+ but not as 3+ (48).

Because of the inside-out growth pattern of neoplastic cell clusters, IMPC often exhibits incomplete membrane staining intensity of *HER2*. Stewart et al. and Perron et al. recommended the incorporation of fluorescence *in situ* hybridization testing to confirm *HER2* amplification (49). However, Zhou et al. demonstrated that if the basolateral membrane showed intense, clear, linear, and incomplete staining of *HER2*, IMPC should be classified as *HER2* 3+ rather than *HER2 2*+. This approach helps avoid the need for additional fluorescence *in situ* hybridization testing procedures, saving considerable time, manpower, and economic costs.

Previously, *HER2* expression was considered clinically insignificant in ductal carcinoma *in situ*; however, Francesk et al. reported that *HER2* over-expression in DCIS is correlated with poorer clinicopathological parameters (50). Moreover, a clinical study showed that trastuzumab treatment in high-risk patients with *HER2*-positive DCIS reduced the recurrence rate (51). There are no studies on whether HER2 expression affects the prognosis of patients with IMPC; thus, this data need to be further explored.

## Clinical examination of IMPC

IMPC exhibits distinct characteristics in different radiological examinations (52–55). On mammography, most lesions are irregular or lobulated, with vague or spiculated margins and scattered calcifications. On ultrasonography, lesions mostly demonstrate longitudinal growth, internal hypoechoic appearance, irregular shape, lobulated margins, and echogenic posterior features. Lesions observed on magnetic resonance imaging (MRI) typically have irregular shapes with spiculated margins. They exhibit heterogeneity in contrast enhancement and demonstrate a type III time-signal intensity curve. On positron emission tomography-computed tomography (PET-CT), the mean maximum standardized uptake value (SUVmax) of the lesions is >10, which distinguishes it from other breast cancer subtypes (**Table 1**).

**Table 1:** Imaging features of invasive micropapillary carcinoma.

Feature	Mammography	Ultrasonograpgy	MRI	PET-CT
Shape	Irregular/Lobulated	Hypoechoic	Irregular	–
Margin	Vague/Spiculated	Lobulated	Spiculated	–
Calcification	Scattered	–	–	–
Posterior feature	–	Hypoechoic	–	–
Enhancement	–	–	Heterogeneous	–
TIC	–	–	Type III	–
SUVmax	–	–	–	>10

TIC, time-signal intensity curve; SUVmax, maximum standardized uptake value; MRI, magnetic resonance imaging; PET-CT, positron emission tomography-computed tomography.

Kubota et al. demonstrated that IMPC is particularly specific in MRI examination and is easy to identify and that ultrasound examination has high sensitivity for LNM of IMPC (56). However, the mammographic and ultrasound imaging characteristics of IMPC are reported to be difficult to distinguish from those of other breast cancer subtypes (57). Moreover, among the 29 cases enrolled in Akgun et al.’s study (58), 13 (13/29,44.82%) had MP component <75% and 16 (16/29,55.18%) had MP component >75%. The data revealed no significant correlation between the SUVmax of PET-CT and the proportion of MP component.

Although IMPC is highly invasive, sentinel lymph node biopsy (SLNB) remains the gold standard for determining the need for axillary lymph node dissection. To date, indocyanine green (ICG) is considered to have a good application prospect in SLNB for IMPC (59).

## Clinical features of IMPC

The median age at IMPC onset is 48.0–61.4 years, with a median diameter of approximately 2.0–5.0 cm (60–62), consistent with the previous findings of Fu et al. (38). However, another study suggests that IMPC tends to be larger than IDC (63).

Liu et al. reported that IMPC had a significantly higher rate of estrogen receptor (ER) positivity (*P* = 0.037) and LVI (*P* = 0.005) than matched IDC (64). Mercogliano et al. further explained that ER expression in IMPC was positively correlated with tumor size but inversely correlated with patient age (65). Yu et al. found that IMPC had a higher nuclear grade III ratio than IDC by comparing the clinicopathological features between 267 IDC and 267 IMPC cases (*P* < 0.001) (66). Aker et al. reported that IMPC is more likely to have LNM than IDC (67). The presence of MP features increases the probability of LNM, LVI, and poor nuclear differentiation. This is closely related to the disordered arrangement and polarity reversal of neoplastic cell clusters (**Table 2**).

**Table 2:** Pathological characteristics between IMPC and IDC.

Author	Characteristic	Relevance	*P*-value
Liu et al. (61)	ER	Positively correlated with IMPC	*P*=0.037
	LVI	Positively correlated with IMPC	*P*=0.005
Mercogliano et al. (62)	ER	Positively correlated with IMPC SIZE	*P*=0.014
Yu et al. (63)	Nuclear grade III	Positively correlated with IMPC	*P*<0.001
Aker et al. (64)	LNM	Positively correlated with IMPC	*P*<0.001

IMPC, invasive micropapillary carcinoma of the breast; IDC, invasive ductal carcinoma of the breast; ER, Estrogen Receptor; LVI, Lymphovascular Invasion; LNM, Lymph Node Metastasis.

Eren et al. found that the positive rate of progesterone receptor (PR) in the pure IMPC group was significantly lower than that in the mixed IMPC group (66.7% vs. 83.3%, *P* = 0.024) (31). Further analysis by Wang et al. revealed a significantly higher proportion of stage IIIc cases in the pure IMPC group than in the mixed IMPC group (38.3% vs. 17.8%, *P* = 0.003) (68). However, Kaya et al. reported no significant difference between pure and mixed IMPC in terms of PR status, tumor size, LNM, LVI, and histological grade (69). Combined with the relevant literature, the incidence of pure IMPC was only 1%–2% (62, 70). The variation in the results among the aforementioned studies may be attributed to the relatively small number of pure IMPC cases, potentially introducing bias (**Table 3**).

**Table 3:** Pathological characteristics between Pure and Mixed IMPC.

Author	Characteristic	pIMPC, n (%)	mIMPC, n (%)	*P*-value
Eren et al. (42)	PR	30 (66.7)	85 (83.3)	*P*=0.024
Wang et al. (65)	IIIc	18 (38.3)	16 (17.8)	*P*=0.003
Kaya et al. (66)	PR	17(89.50)	17(60.70)	*P*=0.188
	Tumor size			
	<2 cm	4(14.3)	2(10.5)	*P*=0.54
	2~5 cm	20(70.4)	16(84.2)	
	>5 cm	1(5.3)	4(17.3)	
	histological grade			
	I	1(3.6)	2(10.5%)	*P*=0.466
	II	18(64.3)	8(42.1%)	
	III	8(28.6)	9(42.1%)	

IMPC, invasive micropapillary carcinoma of the breast; pIMPC, pure invasive micropapillary carcinoma of the breast; mIMPC, mixed invasive micropapillary carcinoma of the breast; PR, progesterone receptor.

In their retrospective analysis, Verras et al. found that genomic sequencing was more likely to detect the luminal B subtype in IMPC than the immunohistochemical method (71). Furthermore, although the triple-negative subtype is less common in IMPC, it is associated with higher histological grade and more advanced disease stage in pathological diagnosis (72–74), indicating that the triple-negative subtype of IMPC is more aggressive. Upon diagnosis, surgery or neoadjuvant therapy is necessary to prevent rapid disease progression.

## Biological hallmark of IMPC

Biological hallmarks are important for diagnosis, treatment information, and predicting the prognosis of IMPC of the breast. Nassar et al. found that mucin1 (MUC1), a glycoprotein encoded by *MUC1* on chromosome 1q21, also known as EMA, is normally expressed on the apical surface of glandular epithelial cells. It is crucial for maintaining lumen formation (75). In IDC, *MUC1* was mainly expressed in the cytoplasm, intercellular space, and apical regions. However, in IMPC, *MUC1* was predominantly expressed on the basal surface of the cells, forming a prominent linear staining band. This band highlighted the outline of the micropapillary structure, providing a basis for diagnosis. *MUC1* has antiadhesive and immunosuppressive properties and can protect against infections. These attributes also position MUC1 as a potential therapeutic target for IMPC (76).

Notably, the staining patterns of sialyl Lewis X (sLeX), *MUC4*, β1 integrin, and *RAC1* reflect the polarity reversal characteristic of IMPC and serve as diagnostic markers for IMPC. Their positive expression is closely related to poor prognosis among patients with IMPC (42, 65). However, Sozzani et al. reported that sLex expression was not a prognostic factor of IMPC (77). Song et al. reported that high sLex expression on the tumor cell membrane in IMPC was associated with shorter overall survival (OS) and disease-free survival (DFS) (*P* = 0.030, *P* < 0.001, respectively) (42). Mercogliano et al. demonstrated that high MUC4 expression was associated with shorter DFS in patients with *HER2*-overexpressing IMPC (*P* = 0.019) (65). Liu et al. showed that overexpression of β1 integrin and *RAC1* was associated with LNM and shorter DFS in IMPC (78).

CD44, a cell surface transmembrane glycoprotein, is involved in tumor cell differentiation, invasion, and metastasis. Badyal et al. showed that the loss of CD44 in IMPC is correlated with LNM, indicating its potential as a marker to predict LNM. This phenomenon is attributed to the re-expression of CD44 in lymph nodes, which guides the homing of tumor cells to lymph nodes. Umeda et al. validated this finding (79, 80).

Meng et al. (81) reported that high expression of prostate stem cell antigen (PSCA), located on chromosome 8q24 and a glycosylphosphatidylinositol-anchored cell surface protein, was significantly associated with shorter DFS among patients with IMPC (*P* = 0.0003). Liu et al. subsequently reported that Jagged1 expression in IMPC was linked to large tumor size, LVI, and Ki67, and it emerged as an independent prognostic factor for DFS and OS (63).

AT-rich interaction domain 1A (*ARID1A*), a novel tumor suppressor gene, is a part of the multiprotein SWI/SNF chromatin remodeling complex and plays an important role in inhibiting the proliferation, differentiation, and invasion of tumor cells. Onder et al. reported that low *ARID1A* expression was a predictor of shorter OS and DFS in IMPC (HR = 15.9, 95% CI: 3.5–71.5, *P* < 0.0001; HR = 7.2, 95% CI: 2–25.4, *P* = 0.002). Additionally, *ARID1A* expression exhibits a similar trend in both pure and mixed IMPC (82).

**Figure 2:**
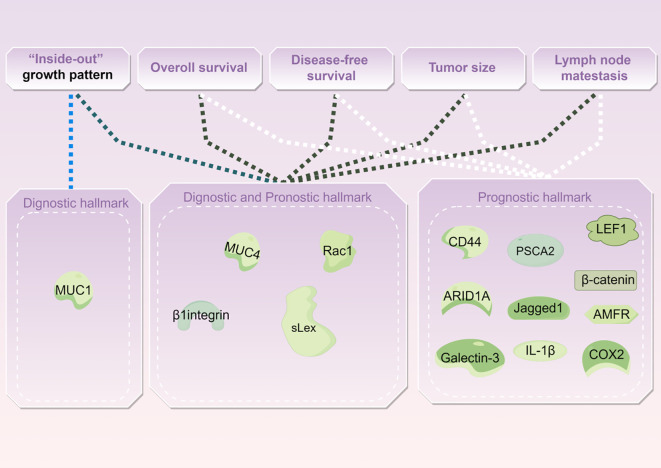
Profiles of biological hallmarks associated with IMPC.

Moreover, cyclooxygenase-2 (*COX-2*), lymphoid enhancer-binding factor 1 (*LEF1*), β-catenin, galectin-3, interleukin-1beta (*IL-1β*), and autocrine motility factor receptor (*AMFR*) were associated with poor prognosis in IMPC (46, 83–87) (**Table 4** and **Figure 2**).

**Table 4:** Biological hallmarks associated with IMPC.

Hallmark	Role	Function	Significance	Ref
*MUC1*	Diagnostic	Maintain lumen integrity in normal glandular tissues	Identify “inside-out” growth pattern	(72)
*sLex*	Diagnostic Prognostic	Associat with the reversal of cell polarity	Identify “inside-out” growth pattern; Shorter OS and DFS	(41)
*MUC4*	Diagnostic Prognostic	confer antiadhesive properties	Identify “inside-out” growth pattern; Shorter DFS	(62)
*β1integrin*	Diagnostic Prognostic	maintain polarity of normal epithelial cells	Identify “inside-out” growth pattern; Shorter OS and LNM	(75)
*Rac 1*	Diagnostic Prognostic			
*CD44*	Prognostic	Reduce adhesion between cell-cell, and cell-basement membrane	LNM	(76, 77)
*PSCA*	Prognostic	Associat with cell adhesion molecules	Shorter OS	(78)
*Jagged1*	Prognostic	Modulate TAMs differentiation	Tumor Size, LVI, and Ki67	(60)
*ARID1A*	Prognostic	Tumor suppressor	Shorter OS and DFS	(79)
*COX-2*	Prognostic	Proliferation, mutagenesis, angiogenesis, etc	Higher histological grade, PR negative, high KI67 expression, and LNM	(45, 80–84)
*LEF1*	Prognostic	Wnt pathway activator		
*β-catenin*	Prognostic	Wnt pathway receptor		
*Galectin-3*	Prognostic	Influence tumor progression and cell polarity		
*IL-1β*	Prognostic	Increase microvessel density		
*AMFR*	Prognostic	Alter cellular adhesion, proliferation, motility, and apoptosis		

TAMs, Tumor-associated macrophages; OS, Overall Survival; DFS, Disease-Free Survival; LNM, Lymph Node Metastasis; LVI, Lymph Node Metastasis.

## Underlying pathogenesis of IMPC

**Figure 3:**
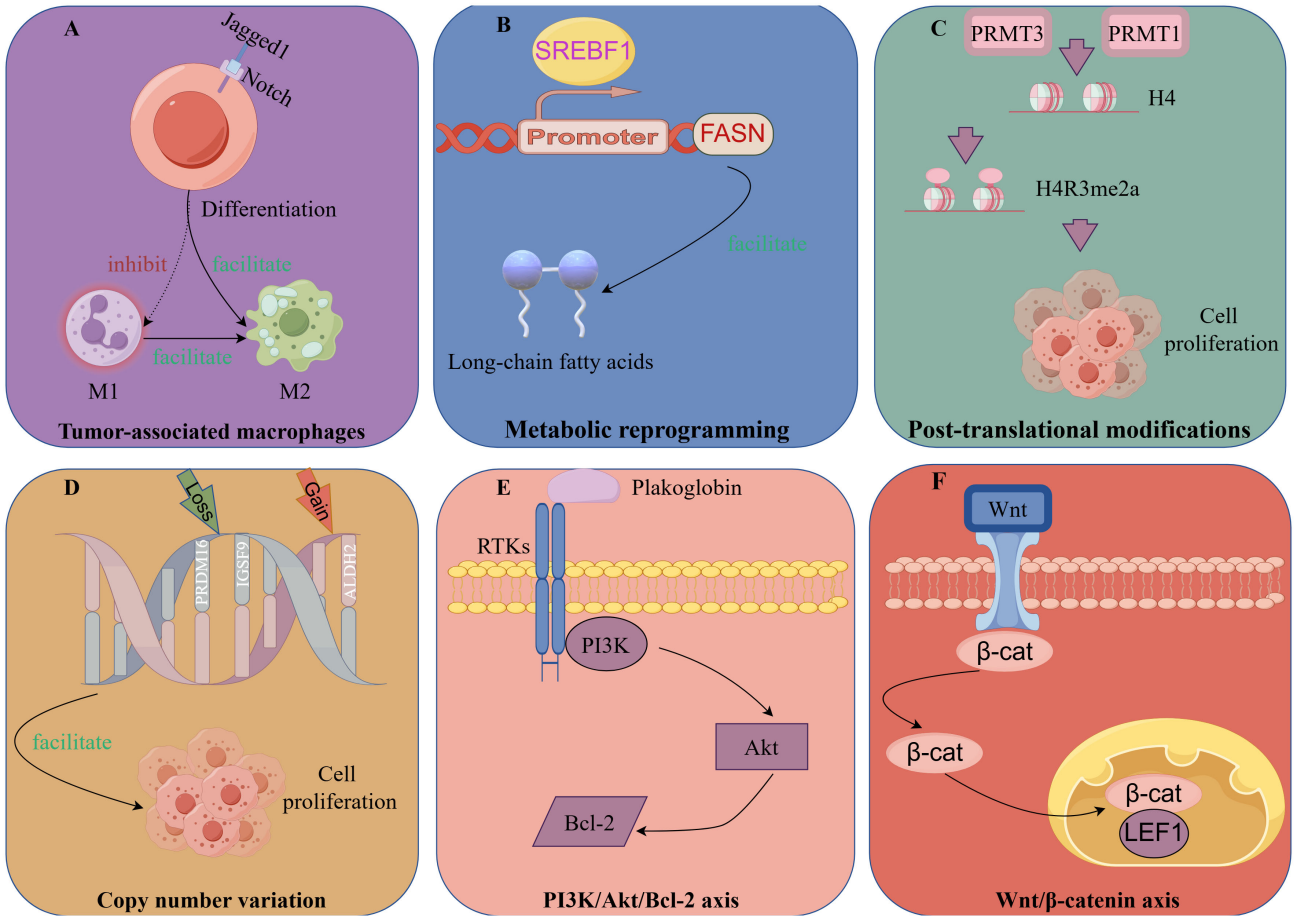
Potential pathogenesis of IMPC.

The key to reducing the high-risk biological behavior of IMPC lies in obtaining a thorough understanding of its pathogenesis and developing individualized treatment approaches for patients with IMPC. In recent years, with advances in research, the onset of IMPC is linked to tumor cell polarity and the tumor-immune microenvironment, including tumor-associated macrophages (TAMs), metabolic reprograming, post-translational modification (PTMs), related signaling pathways, genomic mutations, and copy number variations (CNV) (**Figure 3**).

### Tumor cell polarity of IMPC

The cell polarity reversal pattern is a unique morphological feature of IMPC and is associated with poor prognosis. Uncovering its underlying molecular biological mechanism has become a topic of interest. According to Gruel et al. (88), *LIN7A* was identified as one of the most differentially expressed genes in IDC and IMPC via genome sequencing. *LIN7A* overexpression in IMPC was further confirmed at the mRNA and protein levels. Meanwhile, *LIN7A* overexpression caused cell polarity reversal and tube formation in MCF-10A and MDA-MB-231 3D cell culture models. Additionally, the experimental results demonstrated that tumor cells exhibiting reversed cell polarity displayed stronger proliferation, invasion, and metastatic abilities than those with normal polarity. To our knowledge, this is the first report highlighting the significant role of *LIN7A* in the regulation of tumor cell polarity. However, its upstream and downstream regulatory mechanisms are yet to be explored.

In the study by Liu et al. (78), *RAC1* overexpression induced the reversal of tumor cell polarity and affected the prognosis of patients with IMPC. Following the silencing of the β1 integrin gene via RNA interference (RNAi), there was a significant downregulation in β1 integrin expression and *RAC1* expression. In contrast, inhibition of *RAC1* expression by RNAi did not downregulate β1 integrin expression, leading to the speculation that β1 integrin positively regulates *RAC1*. Furthermore, AIIB2, a β1 integrin inhibitor, abrogated the reversal of tumor cell polarity induced by *RAC1* overexpression. Therefore, β1 integrin induces the reversal of tumor cell polarity by positively regulating *RAC1* expression, thereby influencing IMPC prognosis.

Although *MUC1*, *MUC4*, and sLex are also considered to be associated with tumor cell polarity reversal and poor prognosis, further in-depth molecular mechanisms have not been reported (42, 65, 75).

### Tumor microenvironment of IMPC

#### TAMs

TAMs, important mediators of tumor growth, are functionally categorized into two contrasting subtypes, classical activated M1 macrophages and alternatively activated M2 macrophages (89). The former typically exerts antitumor effects, whereas the latter can promote the occurrence and metastasis of tumor cells. The distinctive feature of M2 macrophages is the expression of the scavenger receptor *CD163* (90).

TAM differentiation is reported to depend on Notch signaling modulation (91). Upregulation of Notch-1 and its signaling following macrophage activation modulate gene expression patterns, affecting antigen-presenting capacity and cytotoxic activity. Jagged1, a Notch receptor ligand, is also important in the regulation of tumor occurrence and development of tumors (92).

Liu et al. (63) reported that Jagged1 was highly expressed in IMPC compared with IDC and served as an independent prognostic factor for DFS. Jagged1 expression was positively correlated with the infiltration of *CD163*+ M2 macrophages in the tumor stroma.

Moreover, *PJA2*, which regulates the intensity and duration of cAMP signaling via protein kinase A (PKA), enhances the accumulation of ubiquitinated malignant fibrous histiocytoma amplified sequence 1, thereby promoting M1 macrophage polarization and M2 to M1 macrophage transformation (93, 94). Aberrant expression of *PJA2* in IMPC promotes tumor invasion via M2 macrophage polarization (95).

TAMs are hypothesized to be influenced by various factors in the IMPC environment, causing a decrease in M1 macrophages and an increase in the number of M2 macrophages, thereby promoting tumor invasion. Hence, targeting jagged1 and *PJA2* to reduce the formation of M2 macrophages could represent a potential therapeutic approach for IMPC.

#### Natural killer T cells

Kanomata et al. demonstrated that CD1d was expressed at abnormally low levels in IMPC (95). *CD1d* is a lipid antigen that activates natural killer T cells (NKT) by interacting with T-cell receptors on the cell membrane (96). Hix et al. found that low expression of *CD1d* compromised the immune function of NKT toward tumor cells and promoted the metastasis of breast cancer *in vitro* and *in vivo* (97). The decreased expression of *CD1d* in IMPC may enable tumor cells to evade immune system regulation and enhance the metastatic potential of IMPC. Improving the immune surveillance function of NTK may be another effective treatment for IMPC.

### Metabolic reprograming of IMPC

Using spatial transcriptome sequencing, Lv et al. (98) mapped the transcriptional profile of IMPC for the first time. IMPC heterogeneity is associated with metabolic reprograming involving unsaturated fatty acid metabolism, long-chain fatty acid metabolism, amino acid metabolism, carbohydrate metabolism, and glycolysis.

Furthermore, their data revealed that *SREBF1* expression was significantly higher in IMPC clusters than in IDC clusters. Interestingly, *FASN*, a target gene of *SREBF1*, was also highly expressed in IMPC clusters. Increased expression of *SREBF1* and *FASN* is closely related to IMPC survival. SREBF1 is a key transcription factor regulating *FASN* in lipid metabolism. *FASN* is a key enzyme involved in the *de novo* synthesis of long-chain fatty acids (99).

*SREBF1/FASN* affects the heterogeneity of IMPC through lipid metabolism and may be a potential therapeutic target of IMPC.

### Post-translational modifications of IMPC

PTMs induce structural changes in existing proteins to participate in multiple biological processes, including tumor initiation, progression, and invasion (100). There are up to 600 types of post-translational modifications of human proteins (101), with common types including acetylation, methylation, phosphorylation, propionylation, butyrylation, crotonylation, 2-hydroxyisobutyrylation, malonylation, and succinylation.

#### Methylation

Protein arginine methyl-transferases play an important role in arginine methylation and are involved in metabolic reprograming during tumorigenesis (102, 103).

Zhi et al. (104), through a comparison of metabolomic differences between IMPC and IDC, identified aberrant arginine methylation and overexpression of protein arginine methyl-transferases 3 (PRMT3) in IMPC. *PRMT3*, a methylation “writer,” is closely related to arginine methylation. Subsequently, FLAG-tag, CO-IP, and bioinformatics analyses revealed that *PRMT3* interacts with histone H4 to increase H4R3me2a levels and mediates the expression of ER stress-related genes. Meanwhile, *PRMT3* overexpression promoted tumor proliferation, whereas *PRMT3* knockdown inhibited tumor growth *in vitro* and *in vivo*.

Wu et al. (23) found that high expression of protein arginine methyl-transferases 1 (*PRMT1*) was associated with shorter DFS in IMPC. In cellular experiments, *PRMT1* also upregulated H4R3me2a to induce tumor cell proliferation and promote tumor cell metastasis via the tumor necrosis factor signaling pathway.

#### Phosphorylation

Protein phosphorylation, a crucial PTM, occurs mainly on serine, threonine, and tyrosine residues and is a reversible process regulated by kinases and phosphatases. Kinases play a significant role in the growth, migration, and invasion of malignant tumors (105, 106).

Chen et al. (107) analyzed the proteomic and phosphoproteomic characteristics of IMPC by LC–MS/MS. Sequencing data revealed that 589 phosphosites on 479 phosphoproteins were considered to be highly phosphorylated in IMPC, whereas 267 phosphosites on 176 phosphoproteins were down-regulated. Enrichment analysis of differentially phosphorylated proteins through GO and KEGG revealed that upregulated phosphoproteins were primarily associated with enzyme activator activity. Kinase enrichment analysis (KSEA) indicated that cyclin-dependent kinases and p90 ribosomal S6 kinases (RSKs) were highly activated, whereas protein kinase A (PKA) and protein kinase C (PKC) families were significantly inhibited in IMPC. Simultaneously, the tumor-specific mTORC1/S6K2 signaling pathway was significantly activated in IMPC. Unfortunately, despite significant attention to the proteome and phosphoproteome profiles of IMPC, the phosphorylation sites of specific proteins associated with IMPC have not been validated by *in vitro* and *in vivo* experiments.

This is the first in-depth proteomic and phosphoproteomic study to explore the pathogenesis of IMPC. The findings of this study suggest that IMPC mediates the activation of proto-oncogenes or the repression of tumor suppressor genes through protein phosphorylation. This process can influence cell cycle regulation and enhance proliferative growth signals to promote tumorigenesis and rapid progression.

#### Other modifications

PTMs are considered closely related to breast cancer. In triple-negative breast cancer, Krug et al. reported (108) that dysregulation of SIRT3 protein extensively affected mitochondrial acetylation in *BRCA*, leading to increased aerobic glycolysis and impaired tricarboxylic acid cycle. Ding et al. demonstrated that *GPX4* ubiquitination upregulates *EGR1* to induce mitochondrial-mediated apoptosis in triple-negative breast cancer cells (109). Pandkar et al. (110) reported that increased lactate production leads to histone H3 lysine 18 lactylation (H3K18la)-mediated upregulation of c-Myc expression, enhancing the invasion of hormone receptor-positive breast cancer. While the modification omics of acetylation, ubiquitination, and lactacylation of IMPC have not been reported, these modifications may play crucial roles in the heterogeneity of IMPC and serve as potential therapeutic targets for IMPC.

### Genomic mutations and copy number variation

Genomic mutations and CNV are considered to drive tumorigenesis and initiate intratumor heterogeneity (111, 112). As reported by Shi et al. (113), CNV in IMPC were positively correlated with LNM by whole exome sequencing and whole genome sequencing of IMPC samples and paired normal tissues, along with cell cluster sequencing of primary IMPC lesions and paired lymph nodes. *PRDM16* and *IGSF9* copy number losses and *ALDH2* copy number gains were observed in IMPC. Furthermore, COX regression analysis confirmed that low expression of *PRDM16* and *IGSF9* and high expression of *ALDH2* were associated with LNM and poor survival in patients with IMPC.

### Related signaling pathways of IMPC

#### PI3K/Akt/Bcl-2 axis

Plakoglobin, a member of the armadillo protein family, is an important component of adhesion junctions and delaminating bodies and can promote tumor cell aggregation and metastasis (114, 115). Huang et al. (116) found that *in vivo* and *in vitro* data showed that plakoglobin was overexpressed in the cell membrane and cytoplasm of IMPC. Plakoglobin knockout resulted in cluster depolymerization, whereas plakoglobin overexpression activated the PI3K/Akt/Bcl-2 signaling pathway and reduced cluster apoptosis in cell models. Furthermore, plakoglobin knockout inhibited tumor proliferation in animal models.

#### Wnt/β-catenin Axis

Dolezal et al. (46) reported that lymphoid enhancer-binding factor 1 (LEF1) and β-catenin expression were significantly increased in lymph node lesions compared with primary lesions in IMPC. Wnt/β-catenin plays a crucial role in the invasion of malignant tumors, and *LEF1* is a specific marker of this signaling pathway (117, 118). Therefore, *LEF1* overexpression may activate the Wnt/β-catenin pathway and contribute to lymph node tropism in IMPC. However, further basic experiments are required to validate this finding.

## Survival prediction models and prognosis

The survival prediction model integrates the information of clinical, imaging, and pathological characteristics of the disease to establish an evaluation system for predicting the individual prognosis of patients. This model helps clinicians develop individualized treatment measures (119).

Meng et al. enrolled 388 patients diagnosed with IMPC and who underwent surgery between 2013 and 2017. A nomogram revealed that factors such as age, LNM, hormone receptor status, adjuvant radiotherapy, vascular invasion of lymph nodes, and postoperative radiotherapy significantly influenced LRR (120). Chen et al. used the Surveillance, Epidemiology, and End Results (SEER) database to construct a nomogram from 1,885 surgically treated patients with IMPC. They discovered that age ≥62 years at diagnosis, estrogen receptor negativity, and tumor stage were adverse independent factors for OS. Conversely, married patients and those treated with chemotherapy or radiotherapy had longer postoperative survival (121). In the retrospective study by Wang et al. (122), patients with IMPC who underwent breast-conserving surgery had better OS and breast cancer-specific survival (BCSS) than those in the mastectomy group. However, patients in the breast-conserving group had a smaller tumor diameter, fewer LNMs, and higher ER- and PR-receptor positivity. In the retrospective study by Wang et al. (122), breast-conserving surgery demonstrated better OS and BCSS than mastectomy for IMPC. However, patients in the breast-conserving group had smaller tumor diameters, fewer LNMs, and higher hormone receptor positivity. Additionally, Lewis et al. screened 2,660 patients from the US National Cancer Database to construct a COX model. They discovered that the survival time of patients with ≥4 positive lymph nodes was significantly shorter than that of patients with negative lymph nodes (*P* < 0.001).

However, the survival time of patients with 1–3 positive lymph nodes was similar to that of patients with negative lymph nodes (*P* = 0.883), indicating that N2 is an independent prognostic factor for IMPC (123). These studies guide patients with IMPC who exhibit the aforementioned high-risk factors before and after surgery. This approach may include expanding the scope of surgery, intensifying systemic therapy, and increasing the dose of radiotherapy.

The presence of MP component within no special type tumors is a more frequent occurrence, and much deliberation has been made on its clinical significance. Multiple studies have reported an association between the presence of a MP element within a tumor and a poorer prognosis, along with a recurring lymphotropic pattern (39). Chen et al. retrospectively analyzed 100 patients with IMPC and found lower 5- and 10-year OS than those with IDC; however, the reliability of their findings was questionable because of the small number of patients enrolled (34). Li et al. (124) used propensity score matching to eliminate the difference between IMPC and IDC during the screening period and revealed that MP was a favorable prognostic factor. However, more studies have suggested that compared with IDC, IMPC has significantly higher relapse-free and local-regional recurrence-free survival rates, with no difference in OS. The varying outcomes of the aforementioned studies may be attributed to a lack of understanding of IMPC in the past, with numerous cases being misdiagnosed, leading to an underestimation of the disease’s impact. However, as the understanding of IMPC improves, its diagnostic rate is gradually increasing. This enables more cases to be treated effectively at an early stage.

## Conclusions

While most current studies suggest that IMPC does not affect OS, its highly aggressive behavior increases the risk of LNM and local recurrence, significantly affecting patients’ quality of life. Moreover, most of the current studies on IMPC are retrospective analyses, making it challenging to correct biases related to tumor stage, surgical methods, and systemic treatments. As a result, there are significant differences between the findings of these studies. Hence, assessing whether IMPC affects OS requires extensive large-scale prospective studies. From a histological perspective, the “inside-out” growth pattern reflects the external manifestation of IMPC’s high invasiveness. However, from a pathogenesis perspective, IMPC heterogeneity is driven by multiple factors, such as tumor-immune microenvironment, TAMs, and metabolic reprogramming. In this review, we focused on recent advances in the biomarkers, pathogenesis, and survival prediction models of IMPC. Additionally, we aimed to deepen our understanding of tumor heterogeneity, provide valuable insights into potential treatment targets, and identify the underlying mechanisms of IMPC to improve treatment strategies.
